# Research on supply chain resilience mechanism of AI-Enabled manufacturing enterprises -based on organizational change perspective

**DOI:** 10.1038/s41598-025-17138-3

**Published:** 2025-08-25

**Authors:** Xiaochuan Guo, You Chen, Jiaping Xie, Huiyan Wang, Xue Lei

**Affiliations:** 1 Shanghai University, Shanghai, China; 2 Shanghai University of Finance and Economics, Shanghai, China; 3 Xinjiang University of Finance and Economics, Urumqi, Xinjiang China

**Keywords:** Artificial intelligence, Supply chain resilience, Organizational change, Manufacturing, Sustainability, Time series, Systems biology

## Abstract

Artificial intelligence (AI) is fundamentally reshaping supply chain operation modes and innovation paths, which has a significant impact on the development of supply chain resilience. This study utilizes panel data of Chinese A-share listed manufacturing companies from 2013 to 2022 to measure the application of AI through textual analysis and construct firm-level supply chain resilience indicators using factor analysis. The main findings suggest that AI greatly enhances supply chain resilience in the Chinese manufacturing industry, and this result holds in a series of robustness tests. Second, AI improves supply chain resilience through changes in organizational structure and improvements in internal control systems. Further, the impact of AI on supply chain resilience varies by industry characteristics and firms’ position in the supply chain. Finally, the technological maturity and depth of AI application within a firm also affects supply chain resilience differently. This study contributes to the research on the application of AI in supply chain management and the theory of supply chain resilience, as well as provides a theoretical foundation and practical insights for manufacturing firms to enhance their own resilience in the face of increasing global uncertainty and complexity.

## Introduction

In recent years, the global economic landscape has undergone profound adjustments, and manufacturing supply chains universally face unprecedented risks and challenges. Globally, unilateralism and protectionism are intensifying, with some countries promoting the reshaping of the Global Value Chain (GVC) landscape through policies such as “reshoring” and “friendshoring.” Concurrently, escalating technological competition has made data, technology, and supply chain security core concerns for businesses worldwide, leading to a continuous rise in the potential risk of supply chain disruptions^1^.According to McKinsey (2021), firms experience short-term supply chain disruptions approximately once every two years, and major disruptions lasting one to two months every 3.7 years. Over a ten-year period, these events can reduce cumulative pre-tax revenue by as much as 42%. Similarly, the World Economic Forum (2023) reports that severe supply chain disruptions may erode over 40% of corporate profits in each decade. These statistics underscore the growing uncertainty within global supply chains and its profound implications for enterprise stability. In this context, how to enhance supply chain resilience and strengthen risk resistance capabilities has become a critical issue that manufacturers and policymakers worldwide urgently need to address collaboratively. For China’s manufacturing sector, this trend likewise presents profound challenges and pressures for transformation. Improving supply chain resilience and bolstering risk resistance are of great significance for preventing risks such as industrial chain fragmentation, supply chain control, and value chain obstruction, thereby ensuring the nation’s initiative in global industrial competition.

In the era of the digital economy, the rapid advancement of artificial intelligence (AI) and other emerging technologies has offered new opportunities for enhancing supply chain resilience in the manufacturing sector. Leveraging its powerful capabilities in data analysis, forecasting, and intelligent decision-making, AI can help enterprises better navigate complex and volatile external environments, optimize resource allocation, and improve collaborative efficiency^2^.However, compared to developed countries, the adoption of AI in Chinese manufacturing firms remains relatively limited. According to a McKinsey report, while the global average AI adoption rate stands at 50%—with the United States reaching 60%—Chinese manufacturing firms lag behind at just 41%. This disparity suggests that Chinese enterprises must act within a limited window of opportunity to upgrade their technologies and accelerate the transition toward intelligent supply chains, or risk falling behind in the global competitive landscape.Moreover, the deeper integration of AI is driving profound changes in the organizational structures and internal control systems of supply chain enterprises, enhancing their responsiveness and adaptability through more efficient decision-making and cross-functional collaboration^3^. Indeed, organizational change is the core link for technology-driven enterprise capability upgrading. Organizational change theory posits that technological innovation can only be transformed into a company’s sustained competitive advantage through management innovations such as organizational structure, governance systems, and process reengineering^4^. The application of Artificial Intelligence (AI) is not merely a tool upgrade; it also involves multi-dimensional transformation processes such as decentralization of decision-making authority, reorganization of cross-departmental collaboration, and optimization of internal controls. These processes are precisely key to enterprises enhancing their dynamic adaptability and resilience^5^. For example, Haier Group, by introducing an AI-driven supply chain management system, achieved flattened organizational structure reform and process reengineering, enabling various business units to make autonomous decisions and respond quickly to market changes, significantly enhancing supply chain flexibility and risk resistance. Therefore, studying the relationship between AI and supply chain resilience from the perspective of organizational change can reveal the deep-seated mechanisms of AI empowerment, compensating for the limitations of a singular technological perspective.

Existing research has predominantly emphasized the role of AI in enhancing supply chain capabilities and operational efficiency^6^. However, its impact on supply chain resilience remains a subject of debate. Some scholars argue that AI-driven decision-making heavily depends on historical data and often requires human intervention to adapt to extreme or unforeseen scenarios. Over-reliance on AI, they suggest, may impair employees’ flexibility and adaptive capacity, thereby undermining the organization’s ability to cope with unstructured risks and reducing overall resilience^7^. The Artificial Intelligence Infrastructure Alliance further reports that 63% of organizations have incurred losses exceeding $50 million due to failures in AI/ML governance.Conversely, other studies contend that AI and machine learning can enhance supply chain resilience by constructing intelligent decision-making frameworks. By integrating historical data with real-time analytics, AI can support more accurate demand forecasting, risk management, and inventory control^6^. For instance, Amazon’s Supply Chain Optimization Technologies (SCOT) leverages deep learning models to forecast demand and sales for over 400 million products in real time, thereby optimizing inventory allocation and reducing the risks of stock-outs and overstock, which ultimately strengthens the adaptability and resilience of its supply chain.Meanwhile, academic research has increasingly sought to define and measure the foundational dimensions of supply chain resilience. For example, Zhang Shushan and Gu Cheng employed the entropy weight method to evaluate supply chain resilience from the dual perspectives of resistance and recovery capacity^8^. Other studies rely on cross-sectional questionnaire surveys to assess resilience, though such methods are often criticized for being susceptible to the halo effect and lacking a unified theoretical framework to capture the formation and evolution of resilience dimensions. Against this backdrop, this study seeks to clarify the mechanisms through which AI influences supply chain resilience in the context of China’s manufacturing sector. Specifically, it explores these mechanisms through the lens of organizational change, aiming to provide theoretical and empirical insights into the dynamic interaction between AI adoption and supply chain adaptability.

Compared with the existing literature, this study offers several key innovations and marginal contributions:

First, drawing on the theory of technological empowerment and incorporating the lens of organizational change, this study investigates how AI enhances supply chain resilience in the manufacturing sector through two primary channels: organizational structure transformation and improvements in internal control systems. It further reveals that the impact of AI varies significantly depending on industry characteristics and the firm’s position within the supply chain. The research framework and conclusions of this paper offer significant theoretical implications for both emerging economies and global manufacturing enterprises. They provide new insights for companies worldwide to address supply chain uncertainties.

Second, grounded in core capability theory, the study develops a multi-dimensional framework for measuring supply chain resilience, encompassing five key dimensions: redundant resources, financial strength, collaborative relationships, operational capability, and human capital. In parallel, AI adoption is disaggregated into three levels—basic terminology, technological infrastructure, and application practice—allowing the analysis to distinguish between the effects of foundational AI technologies and their practical implementations. This approach not only avoids confounding strategic signaling behaviors by firms, but also offers deeper insights into the layered effects of AI on supply chain resilience.

## Literature review and research hypotheses

### Literature review on supply chain resilience enhancement

Supply chain resilience is commonly defined as the ability of a supply chain to rapidly recover to its original performance level—or achieve a new level of financial, operational, or market performance—after experiencing unexpected or disruptive events^9^.Prior studies suggest that enhancing supply chain resilience largely depends on the internal capabilities of firms, particularly in terms of their abilities to absorb risks and adapt to environmental changes. The absorptive dimension emphasizes minimizing losses during crises, while the adaptive dimension focuses on fostering long-term stability by adjusting supply chain structures and operational processes^10^.Building on these dimensions, researchers have proposed various approaches to resilience enhancement, with particular attention to redundancy strategies, flexibility strategies, and collaborative strategies, as illustrated in Table 1.

**Table 1:** Representative literature on supply chain resilience enhancement strategies.

Strategy	Representative Literature	Key Mechanisms	Advantages and Disadvantages
Redundancy Strategy	10、11、12	Backup Resources, Diversified Supply, Safety Stock	Increased Risk Resistance, but Higher Costs
Flexibility Strategy	13、14、15	Flexible Production, Dynamic Management, Elastic Inventory	Strong Adaptability, but High Management Demands
Collaboration Strategy	16、17	Information Sharing, Joint Decision-Making, Risk Sharing	Synergistic Improvement, but High Mechanism Costs

First, redundancy strategies have been examined as a means of safeguarding supply chain stability by enhancing the system’s capacity to withstand external shocks^11^. Agyabeng et al.^10^ argue that redundancy reduces the likelihood of disruption by embedding spare capacity into the supply chain. For instance, firms may diversify sourcing channels, establish safety stocks, or maintain redundant production facilities to buffer against supplier failures. While such strategies can improve risk resistance, they often come at the cost of increased operational expenses, inventory accumulation, and reduced capital efficiency^12^.

Second, flexibility strategies focus on the supply chain’s ability to respond rapidly to fluctuations in market demand or unforeseen disruptions^13^. In contrast to redundancy, flexibility emphasizes dynamic adaptability, such as reconfigurable production systems and responsive supplier management. Toyota, for example, employs a “lean production + flexible manufacturing” model that allows for rapid adjustment of production schedules in the event of supply chain disruptions, thereby minimizing operational impact^14^. Flexibility also extends to pricing and inventory strategies, enabling firms to adjust stock levels and pricing mechanisms in real time based on evolving market signals^15^.

Finally, collaboration strategies emphasize information sharing, joint decision-making, and coordinated risk management among supply chain stakeholders to enhance overall adaptive capacity. Typical models of collaboration include real-time data exchange, integrated crisis response systems, and collective risk-sharing frameworks^16^. For instance, Walmart’s supply chain management (SCM) platform enables real-time sharing of inventory and sales data with suppliers, increasing transparency and mitigating stockouts or overstocks. Similarly, joint purchasing alliances help reduce supplier dependency and resilience by pooling resources and distributing risk across partners^17^.

### Literature review on artificial intelligence and enterprise supply chains

Artificial intelligence, as a core driver of the Fourth Industrial Revolution, is fundamentally transforming supply chain operations and innovation processes, and exerts a profound influence on the development of supply chain resilience^9^. In China, the rapid advancement of big data and AI technologies has accelerated their adoption in large-scale and complex supply chain contexts. However, the application of AI within supply chain enterprises remains at an exploratory stage, largely due to the absence of standardized technical frameworks and mature development paradigms, which has hindered the realization of fully intelligent and systematic practices^18^.Against this background, this study reviews the existing literature from two key perspectives: AI technologies and their applications in supply chain management, as illustrated in Table 2.

**Table 2:** Representative literature on artificial intelligence empowering supply chain resilience.

Hierarchical Classification	Representative Literature	Main Applications/Technologies	Key Conclusions
Technological Level	6、13、19	Machine Learning, Deep Learning, and Computer Vision, etc.	Enhanced Supply Chain Risk Identification Capabilities
Application Level	20、21	Intelligent Scheduling, Image Recognition, and Chatbots, etc.	Improve Resilience and Production Efficiency

At the technological level, artificial intelligence (AI) encompasses a range of computational methods and tools, such as machine learning, deep learning, and computer vision, which enable intelligent analysis and decision-making within supply chain systems. Belhadi et al.^6^ demonstrate that integrating fuzzy logic programming and machine learning algorithms into supply chain management can generate robust decision-making frameworks to assist practitioners in enhancing resilience.In manufacturing supply chains, technologies such as artificial neural networks (ANNs) and support vector machines (SVMs) have been applied to detect and address potentially delayed orders, thereby enabling more accurate risk identification, resource allocation, and prioritization of response strategies^13^. Moreover, advances in computer vision—such as the use of the DETR detection algorithm for defect classification—have achieved detection accuracies of up to 96%, significantly improving quality control processes and enhancing the efficiency of distribution networks^19^.

At the application level, artificial intelligence refers to the practical deployment of AI technologies across real-world supply chain scenarios, including intelligent scheduling, image recognition, and chatbot-based customer services. Based on a survey of 237 manufacturing firms, Lerch et al.^20^ demonstrated that the integration of AI into production systems significantly enhances organizational resilience. This is particularly relevant in today’s manufacturing environment, where market demand is increasingly shifting toward “customized, small-batch” production. In such contexts, intelligent scheduling enables rapid production line reconfiguration, thereby improving production efficiency and reducing operational costs^21^.

A review of the existing literature reveals that although prior studies have investigated the impact of AI on supply chains and their resilience, the absence of standardized technical frameworks and mature implementation paradigms has limited the full-scale adoption of AI in supply chain contexts, keeping it largely in an exploratory phase.

### Research hypotheses

In the context of accelerating industrialization and globalization, supply chain management in the manufacturing sector has become increasingly complex and diversified. Disruptions such as supply chain blockages or breakdowns can have significant and sometimes irreversible effects on the development and transformation of manufacturing enterprises, thereby creating an urgent need for enhanced resilience. This study, grounded in technology empowerment theory, deeply explores the role of AI in building supply chain resilience. Originating from organizational theory and information systems research, technology empowerment theory emphasizes that emerging technologies are not merely tools; rather, they are a force capable of endowing organizations with stronger adaptability and competitiveness by optimizing the acquisition, allocation, and utilization of resources^22^. This theory posits that technology, acting as a catalyst for change, can drive profound transformations in an organization’s internal structure, culture, and capabilities by introducing new tools, processes, and information flows^23^. Such transformations are not only manifested in efficiency improvements but more importantly in the enhancement of organizational adaptability and innovative capacity, enabling them to maintain agility and achieve sustainable development in complex and volatile external environments. For supply chain management, this provides a solid theoretical framework for understanding how AI, through its data analysis, intelligent decision-making, and automation capabilities, reshapes traditional supply chain operating models to build a more resilient supply chain system^24^. As a crucial vehicle for technology empowerment, AI, leveraging its powerful data analysis, intelligent decision-making, and process optimization capabilities, offers entirely new solutions for building supply chain resilience^25^. From the perspective of technology empowerment theory, AI’s enabling effect is primarily realized through two key channels: organizational structure change and internal control change. First, AI facilitates a shift from traditional hierarchical organizational models toward flatter, more collaborative structures. By streamlining vertical hierarchies and reinforcing horizontal integration across departments, AI improves decision-making efficiency and accelerates firms’ responses to supply chain risks. Second, AI enhances internal control systems by optimizing production processes and transforming personnel management practices. These improvements strengthen firms’ capabilities in risk identification, forecasting, and mitigation, thereby reducing the likelihood of supply chain failures and labor-related disruptions. Based on these considerations, this study focuses on the dual channels of organizational restructuring and internal control transformation to examine how AI, as a form of technological empowerment, contributes to the enhancement of supply chain resilience.

First, organizational structure change. Under the guidance of technology empowerment theory, organizational decision-making and departmental collaboration efficiency are considered critical resources for safeguarding supply chain security and enhancing supply chain resilience. Artificial intelligence, as an empowering technology, can optimize the acquisition, allocation, and utilization of these key resources through its deep thinking, intelligent decision-making, and automation capabilities^26^. Specifically, AI will help supply chain enterprises bypass intermediary monitoring activities and automate a large volume of repetitive work and tasks^3^thereby significantly improving organizational decision-making efficiency and collaborative effectiveness. This increase in efficiency and effectiveness is a direct manifestation of the technology empowerment effect at the organizational structure level. Therefore, AI will profoundly influence organizational structure from both vertical and horizontal dimensions, subsequently enhancing supply chain resilience.

From the vertical dimension, traditional supply chain enterprises typically adopt a pyramid-shaped organizational structure, in which departments operate in silos and the inter-firm relationships follow a linear upstream–downstream pattern^27^. This multilayered structure often results in slow decision-making, overdependence on centralized control at the group level, and a lack of responsiveness to external market changes. The hierarchical rigidity—characterized by strictly defined processes, vertical authorization, and horizontal task segmentation—makes it difficult for organizations to coordinate internally and respond promptly to supply chain disruptions. When confronted with unexpected risks, such structural inertia hinders cross-departmental communication and rapid decision-making. The adoption of AI technologies such as machine learning and intelligent automation enables both managers and employees to handle complex tasks more efficiently and reduces reliance on manual oversight. As a result, organizational hierarchies become flatter, decision-making becomes more decentralized, and firms are better positioned to respond quickly to external changes^28^. This not only improves decision-making efficiency but also enables supply chain enterprises to capture risk signals in a timely manner and implement effective countermeasures.

From the horizontal dimension of organizational structure, the application of artificial intelligence is gradually dissolving the boundaries between departments and enhancing the efficiency of interdepartmental collaboration in responding to supply chain risks. In traditional rigid hierarchical structures of supply chain enterprises, horizontal task interdependence among employees is minimal. Each department and individual operates according to a fixed set of rules, and the frequency of interdepartmental communication and interaction is low, leading to slow and restricted information flow^29^.The application of artificial intelligence in supply chain enterprises promotes a shift from highly centralized control to more decentralized decision-making structures. Employees are no longer limited to being mere executors but are increasingly becoming creators, with greater involvement in decision-making and the autonomy to make decisions independently. This transition strengthens horizontal communication among departmental members^30^improves the efficiency of both departmental and organizational collaboration, and ultimately realizes the structural empowerment effect of artificial intelligence on supply chain enterprises. In addition, artificial intelligence technologies applied in departmental approval processes enable automated approvals and related functions. As big data algorithms continue to evolve, the efficiency of collaboration between departments and supply chain units is further improved, encouraging enterprises and departments to establish stronger interconnected “links,” thereby reinforcing the symbiotic relationships among supply chain nodes and enhancing overall supply chain resilience.

Second, changes in the organization’s internal control. Under the framework of technology empowerment theory, the continuous expansion of traditional manufacturing supply chain systems has incorporated an increasing number of external factors. This has not only amplified the difficulty for supply chain enterprises in managing production and personnel but also further weakened their ability to respond to supply chain risks^31^. However, technology empowerment theory posits that emerging technologies can address such challenges by optimizing organizations’ internal control mechanisms. Therefore, the intelligent development of supply chain enterprises, precisely by leveraging artificial intelligence as an empowering technology, facilitates profound transformations in internal control from both production and personnel dimensions, thereby significantly enhancing supply chain resilience.

From the production perspective, the application of artificial intelligence in supply chain enterprises can significantly optimize production processes. With the advancement of informatization and digitization, enterprises have begun to accumulate production-related data; however, according to a research report by Accenture, more than two-thirds of enterprises still lack a clear understanding of how to utilize this data effectively.On the supply side, artificial intelligence applied to production management can analyze and learn from production data, enable real-time control over production stages, and support the self-diagnosis of operational faults. At the same time, AI can simulate virtual production processes based on historical data to forecast raw material procurement needs, demand fluctuations, and inventory levels—thereby ensuring dynamic equilibrium between supply chain production and market demand^32^while also predicting and preventing potential supply chain risks. For example, in high-end equipment manufacturing, AI can mitigate equipment degradation and predict potential downtime by dynamically adjusting workloads. This helps prevent productivity losses caused by equipment failures and wear, and ensures stable production line operations through automated scheduling and condition-based maintenance^33^. On the demand side, in a market increasingly driven by customization, deep learning and other algorithmic technologies can be used to analyze customer preferences and generate accurate demand profiles. This allows supply chain enterprises to meet the needs of long-tail customers at lower costs, thereby mitigating the risk impact of demand fluctuations on supply chain stability.

From the personnel dimension, the application of artificial intelligence can improve the efficiency of personnel management. A major source of internal control problems is personnel—namely, managers and employees who fail to properly implement or who intentionally disregard organizational control policies due to agency conflicts, insufficient training, or fraudulent intent^34^. The “data-intelligence” model, based on human–machine collaboration and automated decision-making, can effectively reduce executive power, thereby limiting the potential for internal control manipulation and decreasing the incidence of internal risks in supply chain enterprises. For employees, traditional centralized organizational structures tend to inhibit their sense of belonging. In supply chain enterprises, the application of robots and intelligent decision-making systems can liberate blue-collar workers from complex and high-risk work environments, while also reducing the programmatic demands placed on white-collar staff. As a result, employees gradually shift from being mere implementers of production instructions to playing the role of “administrators,” realizing the psychological empowerment effect of artificial intelligence within supply chain enterprises. This enhances employees’ sense of belonging, mitigates labor disputes, and improves employer–employee relationships^35^thereby increasing management efficiency and reducing supply chain risks associated with personnel inefficiencies.

Based on the above analysis, this paper proposes the following hypothesis: artificial intelligence in supply chain enterprises enhances supply chain resilience through two mechanisms—organizational structure change and internal control system adjustment.

**Fig. 1:**
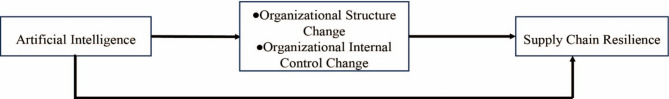
Construct Model.

The construct model is illustrated in Fig. 1.

## Research design

### Sample selection and data sources

This study focuses on Chinese A-share listed companies in the manufacturing industry, covering the sample period from 2013 to 2022. The annual reports of listed firms are obtained from the Sina Finance website (https://finance.sina.com.cn/); patent data are sourced from the China National Intellectual Property Administration (CNIPA); employee data are drawn from the Wind database; and the remaining firm-level data are collected from the CSMAR database. To ensure data availability and reliability, the sample is processed as follows: (1) companies designated as ST, PT, or under insolvency proceedings are excluded; (2) observations with substantial missing values for key variables are removed; and (3) all continuous variables are winsorized at the 1% level on both tails to mitigate the influence of extreme values. After these adjustments, a final sample of 18,617 firm-year observations is obtained.

## Variable definition

### Supply chain resilience (SCR)

Building on prior research, this study incorporates the measurement of supply chain resilience within the framework of core competency theory and constructs it using factor analysis.

To strengthen supply chain resilience, enterprises typically need to integrate and coordinate multiple strategies and capabilities to identify risk nodes, assess risk probabilities, and respond effectively. Core competency theory posits that an enterprise’s core competencies are not tied to a specific product line or department, but rather represent an integrated knowledge system that is difficult for competitors to imitate or acquire. These competencies are developed through long-term investment in production activities and organizational learning^36^.Accordingly, supply chain resilience—defined as a firm’s systematic ability to resist external shocks and recover rapidly—should be viewed as a capability system composed of interrelated and mutually reinforcing components. Based on this understanding, the present study constructs supply chain resilience around the following core dimensions:

- Financial strength: As a fundamental resource supporting supply chain resilience, financial strength enables enterprises to buffer risks and adjust resource allocation in response to uncertain external environments, thereby facilitating production recovery^19^. Existing studies typically use return on net assets and net profit margin on total assets as measurement indicators^37^.
- Redundant resources: As a supplementary element of resilience capacity—such as inventory, spare capacity, and diversified suppliers—redundant resources provide enterprises with the flexibility to respond to unexpected events. When supply chain risks arise, these resources can be converted into actual production capacity to maintain continuity in supply chain operations^13^. Drawing on existing studies, the ratio of administrative expenses to operating income is selected as an indicator to measure a firm’s redundant resource level^38^.
- Operational capacity: The efficient operation of a supply chain enables enterprises to flexibly adjust production and supply strategies in dynamic environments, allowing them to cope with demand fluctuations and potential disruptions—an essential manifestation of supply chain resilience. Existing studies commonly use accounts payable turnover and accounts receivable turnover as measurement indicators^39^.
- Collaboration: As a key component of external supply chain relationships, the supply chain functions as a dynamic alliance involving sellers, suppliers, customers, and other stakeholders. Such collaboration facilitates the sharing of resources, information, and risks across enterprises, thereby strengthening supply chain resilience. Drawing on existing research, supply chain concentration is selected as a measurement indicator^30^.
- Human capital: As a critical driver of supply chain resilience, high-quality human capital—reflected in the skills, knowledge, and experience of enterprise employees—contributes to greater innovation performance and flexibility within the supply chain^3^. It also ensures the continuity of operations and enhances post-disruption recovery capabilities. Existing studies typically use the proportion of employees holding a bachelor’s degree or above as a measurement indicator^40^.

The specific variables are shown in Table 3.

**Table 3:** Definition and measurement of variables.

Variable types and dimensions		Variable name	Variables and measurement
Explained variable	Financial strength	Return on net assets(ROE)	Net profit/shareholders’ equity balance (X1)
		Total Assets Net Profit(ROA)	Net profit/total assets balance (X2)
	Redundant resources	Sinking redundant resources(RS)	Administrative expenses/operating income (X3)
	Operational capacity	Accounts payable turnover(Aptr)	Operating costs/average accounts payable occupancy (X4)
		Accounts receivable turnover ratio(Rtr)	Operating income/average occupancy of accounts receivable (X5)
	Collaborative relationships	Supply chain concentration(Scc)	Average of the sum of the ratio of sales to purchases from the top 5 suppliers and customers (X6)
	Human Resource	Percentage of people with a bachelor’s degree or higher(Degree)	Number of persons with bachelor’s degree and above as a ratio of the total number of persons with bachelor’s degree and above (X7)
Explanatory variable		Artificial Intelligence (AI)	Artificial Intelligence: total number of AI word frequencies
Control Variable		Board Size(Board)	Logarithm of the number of board members
		Firm Age(Firmage)	Logarithm of the time of establishment of the enterprise
		Top 1 Shareholding(Top1)	Ratio of the number of shares held by the largest shareholder of the enterprise to the total number of shares of the enterprise
		Enterprise size(Size)	Logarithm of total assets at the end of the period
		Liability(Lev)	Total assets/total liabilities

### Artificial intelligence (AI)

As enterprises implement AI transformation as a key development strategy, relevant content is often summarized and highlighted in their annual reports. Therefore, it is feasible to apply textual analysis to conduct word frequency statistics based on these reports.

The specific steps are as follows: (1) Data collection: Annual reports of listed manufacturing companies from 2013 to 2022 are obtained from the Sina Finance website, which serves as the primary data source for this study.(2) Artificial intelligence dictionary construction: To build the AI keyword dictionary, this study draws on authoritative sources such as the Artificial Intelligence Index Report 2024 published by Stanford University, the China Artificial Intelligence Industry Market Outlook Research Report 2024 compiled by the China Business Industry Research Institute, and relevant documents from the World Intellectual Property Organization (WIPO). After removing duplicate and irrelevant terms, a total of 37 AI-related keywords are retained, as shown in Table 4.

**Table 4:** Artificial intelligence dictionary.

	Keywords
**AI Terminology**	Artificial Intelligence, Machine Learning, Deep Learning, Natural Language Processing, Computer Vision, Expert Systems, Reinforcement Learning, Neural Networks, Pattern Recognition, Robotics, Data Mining, Automation, Chatbots, Intelligent Control Systems
**AI Technology**	Supervised learning, unsupervised learning, semi-supervised learning, reinforcement learning algorithms, convolutional neural networks, recurrent neural networks, genetic algorithms, support vector machines, clustering algorithms, natural language processing techniques
**AI Applications**	Automated driving, intelligent assistants, healthcare, plagiarism detection, financial fraud detection, image recognition, face recognition, data visualization, product quality monitoring, defect management, intelligent scheduling

Considering that China’s supportive policies for intelligent transformation may encourage enterprises to engage in strategic signaling within annual reports, there is a risk of overstatement or misrepresentation of AI involvement. This often manifests as the excessive use of generic AI-related terminology, even when actual technological deployment or standardized applications are lacking. To account for this, the AI dictionary is further divided into three distinct levels: AI terminology, AI technology, and AI application. This classification enables a more nuanced analysis of how different stages of AI development affect supply chain resilience in China’s manufacturing sector.(3) Construction of AI indicators: The categorized AI dictionary is used as a predefined set of technical terms. The open-source Python module “jieba” is employed to segment the text of each firm’s annual report, and keyword frequencies are calculated accordingly. These frequency counts are then used to construct firm-level AI indicators for subsequent empirical analysis.

### Control variables

To ensure the robustness of the empirical results, this study incorporates several control variables, following the approach of Yao et al.^41^. These include firm size (Size), leverage ratio (Lev), the shareholding ratio of the largest shareholder (Top1), board size (Board), and firm age (Firmage). The definitions and measurements of all main variables are summarized in Table 3.

### Treatment of supply chain resilience variables

#### Applicability and correlation test

The applicability and correlation test is carried out by using KMO test, which is obtained by comparing the correlation coefficient and partial correlation coefficient between the variables, and the results of KMO test and Bartlett’s test are obtained by using IBM SPSS Statistics (Version 26.0, https://www.ibm.com/products/spss-statistics) software, as shown in Table 5, which shows that the KMO statistic are all greater than 0.5, and the P-value of the Bartlett’s spherical test is 0.000, which indicates that the selected variables have strong correlation with each other and are suitable for factor analysis.

**Table 5:** Results of KMO test and Bartlett test for cross-sectional data from 2013 to 2022.

Year		2013	2014	2015	2016	2017	2018	2019	2020	2021	2022
KMO		0.527	0.521	0.524	0.521	0.535	0.563	0.573	0.582	0.598	0.602
Bartlett’s spherical test	Approximate chi-square	2284.922	2279.808	2332.449	2868.684	3744.416	4449.661	5069.394	5173.712	6882.540	7866.563
	df	21	21	21	21	21	21	21	21	21	21
	Sig.	0	0	0	0	0	0	0	0	0	0

#### Factor analysis

As there are some missing parts of the original data, which will affect the results of factor analysis, this paper adopts the linear interpolation method to supplement the missing values by constructing the linear function of the neighboring samples of the missing values to ensure the sample capacity, and further adopts the shrinking-tail treatment to avoid the impact of extreme values. The principal component method is used to extract the common factors, and the resulting eigenvalues and variance contribution rates of the factors are shown in Table 6, the eigenvalues of the first three factors are all greater than 1, and the cumulative variance is basically above and below 65%. Therefore, the three extracted factors basically cover the information of the secondary indicators.

**Table 6:** Contribution rate of total variance in factor analysis of cross-section data from 2012 to 2020.

Year	Component	Initial eigenvalue			Sum of squares loading extraction			Rotated sum of squares loading		
		Total	Variance%	Cumulative%	Total	Variance%	Cumulative%	Total	Variance%	Cumulative%
2013	1	1.956	27.940	27.940	1.956	27.940	27.940	1.915	27.362	27.362
	2	1.584	22.622	50.561	1.584	22.622	50.561	1.602	22.879	50.241
	3	1.054	15.057	65.619	1.054	15.057	65.619	1.076	15.378	65.619
2014	1	1.916	27.370	27.370	1.916	27.370	27.370	1.910	27.279	27.279
	2	1.593	22.760	50.130	1.593	22.760	50.130	1.348	19.254	46.533
	3	1.077	15.379	65.509	1.077	15.379	65.509	1.328	18.976	65.509
2015	1	1.958	27.977	27.977	1.958	27.977	27.977	1.942	27.748	27.748
	2	1.563	22.329	50.306	1.563	22.329	50.306	1.308	18.683	46.430
	3	1.035	14.789	65.095	1.035	14.789	65.095	1.307	18.665	65.095
2016	1	1.978	28.261	28.261	1.978	28.261	28.261	1.875	26.785	26.785
	2	1.454	20.769	49.030	1.454	20.769	49.030	1.547	22.101	48.886
	3	1.046	14.936	63.966	1.046	14.936	63.966	1.056	15.080	63.966
2017	1	2.121	30.307	30.307	2.121	30.307	30.307	1.907	27.236	27.236
	2	1.437	20.525	50.831	1.437	20.525	50.831	1.635	23.355	50.592
	3	1.039	14.848	65.679	1.039	14.848	65.679	1.056	15.087	65.679
2018	1	2.197	31.388	31.388	2.197	31.388	31.388	2.042	29.174	29.174
	2	1.325	18.932	50.320	1.325	18.932	50.320	1.451	20.723	49.897
	3	1.011	14.437	64.756	1.011	14.437	64.756	1.040	14.860	64.756
2019	1	2.244	32.057	32.057	2.244	32.057	32.057	2.125	30.358	30.358
	2	1.311	18.728	50.786	1.311	18.728	50.786	1.423	20.328	50.686
	3	1.034	14.771	65.557	1.034	14.771	65.557	1.041	14.870	65.557
2020	1	2.229	31.849	31.849	2.229	31.849	31.849	2.126	30.368	30.368
	2	1.317	18.811	50.660	1.317	18.811	50.660	1.416	20.226	50.594
	3	1.026	14.653	65.313	1.026	14.653	65.313	1.030	14.719	65.313
2021	1	2.386	34.084	34.084	2.386	34.084	34.084	2.082	29.747	29.747
	2	1.386	19.800	53.884	1.386	19.800	53.884	1.688	24.112	53.859
	3	1.053	15.049	68.933	1.053	15.049	68.933	1.055	15.074	68.933
2022	1	2.395	34.208	34.208	2.395	34.208	34.208	2.100	29.997	29.997
	2	1.406	20.081	54.290	1.406	20.081	54.290	1.696	24.225	54.222
	3	1.064	15.206	69.495	1.064	15.206	69.495	1.069	15.273	69.495

#### Synthesis of the composite index

The scores of the three factors (F1,F2,F3), and the coefficient matrix of the 2020 factor scores are presented in Table 7, and the factor scores of a sample company are calculated based on the coefficient matrix of the 2020 factor scores:

**Table 7:** Factor score coefficients in 2020.

Secondary indicator	Component			Secondary indicator	Component		
	1	2	3		1	2	3
Return on net assets(X1)	0.895	0.311	0.103	Accounts receivable turnover ratio(X5)	0.253	−0.512	−0.006
Total Assets Net Profit(X2)	0.897	0.321	0.080	Supply chain Concentration(X6)	−0.071	−0.073	0.954
Sinking redundant resources(X3)	−0.660	0.219	0.197	Percentage of people with a bachelor’s degree or higher(X7)	−0.151	0.595	0.153
Accounts payable turnover(X4)	0.311	−0.670	0.188				

$$\text{F}1=0.895\text{X}1+0.897\text{X}2-0.660\text{X}3+0.311\text{X}4+0.253\text{X}5-0.071\text{X}6-0.151\text{X}7$$

$$\text{F}2=0.311\text{X}1+0.321\text{X}2+0.219\text{X}3-0.670\text{X}4-0.512\text{X}5-0.073\text{X}6+0.595\text{X}7$$

$$\text{F}3=0.103\text{X}1+0.080\text{X}2+0.197\text{X}3+0.188\text{X}4-0.006\text{X}5+0.954\text{X}6+0.153\text{X}7$$

Subsequently, based on the variance contribution rate of each public factor, the composite factor score (Scr) of supply chain resilience of the sample company is derived. Taking 2020 as an example, the Scr of a sample company is calculated as follows:

$$\text{Scr}\:= (31.849\%\times \text{F}1+18.811\%\times \text{F}2+14.653\%\times\text{F}3) /65.313\%$$

In accordance with the above methodology, the Supply Chain Resilience (Scr) composite index scores of each listed company in each year were calculated as a measure of supply chain resilience.

### Descriptive statistics

Table 8 reports the descriptive statistics of the main variables, with a mean of 5.3261 and a standard deviation of 5.055 for supply chain resilience (SCR), indicating that the level of supply chain resilience varies considerably across enterprises. The standard deviation and mean of Artificial Intelligence (AI) are 29.024 and 11.2819, respectively, indicating that there are obvious differences between the strength and results of AI transformation carried out by enterprises, and that the degree of integration between AI and the real economy is uneven, and that the horizontal differences of the rest of the control variables are more significant, and that all the variables are within a reasonable range.

**Table 8:** Descriptive statistics.

Variable	Sample size	Mean	std	Min	Median	Max
SCR	18,617	5.3261	5.055	−11.06	24.56	SCR
AI	18,617	11.2819	29.024	0.00	587.00	AI
Size	18,617	22.1407	1.186	17.64	27.62	Size
Lev	18,617	0.3956	0.192	0.01	2.47	Lev
Top1	18,617	32.9007	14.140	1.84	89.99	Top1
Board	18,617	2.1037	0.190	1.39	2.89	Board
FirmAge	18,617	2.9485	0.301	1.61	4.17	FirmAge

### Model setting

To test the impact of AI on manufacturing supply chain resilience, this paper sets up the following regression model (1):

1 $$SC{R_{i,t}}=\alpha +\beta A{I_{{\text{i}},t}}+\gamma C{\text{ontrol}}{{\text{s}}_{{\text{i}},t}}+{\mu _i}+{\eta _{\text{t}}}+{\varepsilon _{it}}$$

In model (1), subscripts i and t denote company and year, respectively, and the explanatory variable SCR stands for supply chain resilience; AI is an artificial intelligence indicator, which is obtained based on the AI word frequency statistics of corporate annual reports; Controls is a series of control variables affecting the resilience of the supply chain, and $\:{\mu\:}_{i}$ and$\:\:{\eta\:}_{t}\:$are the industry and year fixed effects, respectively. $\:{\epsilon\:}_{\text{it}}\:$is the model random error term.

## Empirical results and analysis

### Benchmark regression

Table 9 presents the regression results examining the impact of AI on supply chain resilience in the manufacturing sector. The coefficients of the AI variable are significantly positive across columns (1) through (4). Specifically, column (1) reports baseline results without any control variables or fixed effects. Column (2) includes control variables, while column (3) incorporates year and industry fixed effects. Column (4) includes both control variables and fixed effects. In all specifications, the coefficients remain significantly positive at the 1% level, providing preliminary evidence that AI plays a significant role in enhancing supply chain resilience among manufacturing firms.

**Table 9:** Benchmark regression results.

	(1)	(2)	(3)	(4)
	SCR	SCR	SCR	SCR
AI	0.00386^***^	0.00349^***^	0.00648^***^	0.00671^***^
	(3.02)	(2.75)	(6.66)	(6.92)
Size		−0.125^***^		−0.0355
		(−3.47)		(−1.30)
Lev		−1.540^***^		−2.062^***^
		(−7.16)		(−12.66)
Top1		0.00116		−0.000230
		(0.44)		(−0.12)
Board		0.966^***^		0.0199
		(4.84)		(0.13)
FirmAge		−1.720^***^		−0.0302
		(−13.88)		(−0.30)
_Cons	5.283^***^	11.66^***^	5.253^***^	6.908^***^
	(132.93)	(14.20)	(180.58)	(10.76)
Industry	No	No	Yes	Yes
Year	No	No	Yes	Yes
N	18,617	18,617	18,617	18,617
R^2^	0.000491	0.0189	0.473	0.479

Note: Numbers in parentheses are t-values, ***, **, * indicate significant at 1%, 5%, and 10% confidence levels, respectively (same below).

### Endogeneity and robustness test

(1) Instrumental variable approach. Given that higher supply chain resilience in manufacturing firms often reflects stronger internal resource endowments and more stable supply chain relationships, such firms may also be more capable and motivated to adopt AI technologies, thus giving rise to potential reverse causality. To address this issue, this study employs the one-period lag of AI (AI_IV) as an instrumental variable. The regression results are presented in Table 10. The results from both the first-stage and second-stage regressions indicate that AI remains significantly positive at the 1% level, with estimated coefficients of 0.969 and 0.006, respectively. The Anderson LM statistic yields a p-value of 0.000, suggesting that the model does not suffer from under-identification. Furthermore, the Cragg-Donald Wald F-statistic substantially exceeds the critical value proposed by Stock and Yogo, confirming that weak instrument concerns are not present. These results further support the robustness of the paper’s conclusions.

**Table 10:** Endogeneity and robustness tests.

	(1)	(2)	(3)
Variables	First stage	Second stage	Heckman
AI_IV	0.969***		
	(408.02)		
AI		0.006***	0.00672^***^
		(5.73)	(6.93)
IMR			16.05^*^
			(1.66)
Size	0.075	−0.045	0.439
	(1.13)	(−1.52)	(1.53)
Lev	−0.220	−1.867***	−4.345^***^
	(−0.54)	(−10.43)	(−3.13)
Top1	−0.001	0.002	−0.0245^*^
	(−0.20)	(0.85)	(−1.66)
Board	0.534	−0.058	−6.464^*^
	(1.46)	(−0.36)	(−1.65)
FirmAge	−0.327	0.133	1.371
	(−1.28)	(1.18)	(1.61)
Constant	−1.377	6.678**	−5.789
	(−0.23)	(2.54)	(−0.75)
Industry	Yes		Yes
Year	Yes		Yes
N	15,544	15,544	18,617
R^2^	0.9226	0.481	0.479

(2) Heckman test. To address potential endogeneity arising from sample self-selection—particularly selection bias—this study employs the Heckman two-step procedure. In the first stage, a binary variable is constructed based on the median value of AI word frequency: firms with values greater than or equal to the median are coded as 1, and those below as 0. This binary outcome is regressed on firm size, leverage ratio, the shareholding ratio of the largest shareholder, board size, and firm age using a Probit model. From this regression, the inverse Mills ratio (IMR) is calculated. In the second stage, the IMR is included as an additional explanatory variable in the baseline regression (Model 1). The results, reported in column (3) of Table 10, show that the coefficient of AI remains significantly positive at the 1% level, while the IMR is also significant at the 10% level. These findings confirm the presence of selection bias and the necessity of the Heckman correction, thereby further validating the positive effect of AI on supply chain resilience.

(3) Replacement of explanatory variables. Following the approaches of Yao et al.^41^this study remeasures AI levels using two alternative indicators—AI-related keyword frequency and the number of AI-related patents—based on Model (1). The corresponding results are reported in columns (1) and (2) of Table 11. The estimated coefficients are 0.00453 and 0.00657, respectively, and both are statistically significant at the 1% level, thereby reaffirming the validity of the core hypothesis.

**Table 11:** Robustness test results.

	(1)	(2)	(3)	(4)	(5)	(6)
	SCR	SCR	SCR	SCR	SCR	SCR
AI	0.00453^**^	0.00657^***^	0.00772^***^	0.00586^***^	0.00597^***^	0.00601^***^
	(2.48)	(3.58)	(8.57)	(6.02)	(6.20)	(5.71)
Size	−0.0512^*^	−0.0437	−0.0484^*^	−0.0750^***^	−0.0748^***^	−0.0441
	(−1.84)	(−1.60)	(−1.90)	(−2.73)	(−2.75)	(−1.50)
Lev	−2.020^***^	−2.029^***^	−1.836^***^	−2.053^***^	−2.020^***^	−1.868^***^
	(−12.39)	(−12.45)	(−12.04)	(−12.58)	(−12.43)	(−10.42)
Top1	0.00000426	0.0000310	0.000141	0.000282	−0.000301	0.00183
	(0.00)	(0.02)	(0.08)	(0.14)	(−0.15)	(0.85)
Board	0.0211	0.0334	0.0339	−0.00373	0.0110	−0.0544
	(0.14)	(0.22)	(0.24)	(−0.02)	(0.07)	(−0.34)
FirmAge	−0.0697	−0.0435	−0.0687	−0.0664	−0.0854	0.131
	(−0.70)	(−0.44)	(−0.75)	(−0.67)	(−0.87)	(1.16)
_cons	7.409^***^	7.120^***^	7.164^***^	7.928^***^	7.954^***^	6.664^***^
	(11.35)	(11.09)	(12.00)	(12.21)	(12.36)	(9.50)
Industry	Yes	Yes	Yes	Yes	Yes	Yes
Year	Yes	Yes	Yes	Yes	Yes	Yes
Industry* Year	No	No	Yes	No	No	No
Pro	No	No	No	Yes	No	No
Pro* Year	No	No	No	No	Yes	No
N	18,617	18,617	18,617	18,617	18,617	15,544
R^2^	0.478	0.478	0.562	0.489	0.508	0.481

(4) Controlling for annual and regional effects. To further mitigate the confounding effects of time-varying events, column (3) of Table 11 includes industry-year interaction terms to absorb shocks from industry-specific annual events. The coefficient remains significantly positive at 0.00772 (1% level). Column (4) adds controls for regional fixed effects, while column (5) introduces region-year interaction terms to account for regional heterogeneity and external shocks related to regional development differences. The estimated coefficients in columns (4) and (5) are 0.00586 and 0.00597, respectively, both significant at the 1% level, once again confirming the robustness of AI’s positive effect on supply chain resilience.

(5) Lagged explanatory variable test. Since annual reports not only summarize past performance but also outline future strategic plans, the AI-related word frequency derived from these reports may not be immediately reflected in current-year business operations. Therefore, this study re-estimates the model using a one-period lag of the AI variable. The results, shown in column (6) of Table 11, yield a regression coefficient of 0.00601, which remains significantly positive at the 1% level. These findings are consistent with the results from Model (1), further supporting the causal link between AI and enhanced supply chain resilience.

## Mechanism testing

Based on the preceding theoretical analysis, this section empirically examines the impact of AI on manufacturing supply chain resilience through two primary mechanisms: organizational structure change and organizational internal control change.

(1) Organizational structure change channel. Drawing on the work of Li et al.^28^uses the change in the proportion of senior executives as a proxy variable for organizational structure change. When enterprises promote organizational structural change, they often adjust executive levels and numbers to adapt to new strategic goals and environmental shifts. Especially during digital or AI transformation, enterprises frequently need to optimize decision-making processes, strengthen cross-departmental collaboration, or implement flatter management structures. These changes are typically accompanied by dynamic adjustments in the proportion of senior executives^42^. For example, a company might increase the number of executives with digital or technological backgrounds to lead innovation or streamline management layers to improve decision-making efficiency. Therefore, variations in the proportion of senior executives can, to some extent, reflect the process of organizational structural adjustment and optimization of the power and responsibility system within an enterprise, making it a highly operable quantitative indicator for measuring organizational structure change. Since a flatter structure typically means a smaller relative proportion of managers, this is a negative indicator. Consequently, this paper selects the ratio of senior executives to the total number of employees (Ctr) to measure organizational structure change. As shown in column (1) of Table 12, the coefficient of AI is − 0.00718, which is statistically significant at the 5% level, suggesting that AI contributes to supply chain resilience by facilitating organizational flattening. To further validate this channel, column (2) investigates the direct impact of organizational structure on supply chain resilience, and column (3) includes the structure variable as a control in Model (1). The results consistently indicate that AI improves supply chain resilience by promoting structural flattening within manufacturing enterprises.

**Table 12:** Mechanism test regression results.

	(1)	(2)	(3)	(4)	(5)	(6)
	Ctr	SCR	SCR	IC	SCR	SCR
AI	−0.00718^**^		0.00668^***^	0.134^***^		0.00660^***^
	(−1.97)		(6.89)	(3.99)		(6.81)
Ctr		−0.00415^**^	−0.00395^**^			
		(−2.12)	(−2.02)			
IC					0.000846^***^	0.000804^***^
					(3.98)	(3.79)
Size	−3.492^***^	−0.0525^*^	−0.0493^*^	29.27^***^	−0.0628^**^	−0.0591^**^
	(−34.04)	(−1.86)	(−1.75)	(30.97)	(−2.24)	(−2.11)
Lev	−3.575^***^	−2.046^***^	−2.076^***^	−167.7^***^	−1.889^***^	−1.927^***^
	(−5.85)	(−12.54)	(−12.73)	(−29.78)	(−11.33)	(−11.56)
Top1	−0.0466^***^	−0.000356	−0.000414	0.823^***^	−0.000860	−0.000892
	(−6.29)	(−0.18)	(−0.21)	(12.07)	(−0.43)	(−0.45)
Board	1.037^*^	0.0235	0.0240	−2.693	0.0215	0.0221
	(1.84)	(0.16)	(0.16)	(−0.52)	(0.14)	(0.15)
FirmAge	−1.089^***^	−0.0668	−0.0345	−24.23^***^	−0.0414	−0.0107
	(−2.93)	(−0.67)	(−0.35)	(−7.06)	(−0.42)	(−0.11)
_cons	86.87^***^	7.481^***^	7.251^***^	97.28^***^	7.036^***^	6.830^***^
	(36.04)	(11.26)	(10.92)	(4.38)	(10.95)	(10.63)
Industry	Yes	Yes	Yes	Yes	Yes	Yes
Year	Yes	Yes	Yes	Yes	Yes	Yes
N	18,617	18,617	18,617	18,617	18,617	18,617
R^2^	0.148	0.478	0.479	0.102	0.478	0.480

(2) Organizational internal control change channel. A robust internal control system not only helps businesses standardize operational processes and strengthen risk prevention, but also enhances an organization’s ability to respond to external shocks (1). The application of Artificial Intelligence (AI) technology accelerates enterprise process automation and information integration, which helps drive profound changes in internal control across areas like system development, process execution, and risk identification. On one hand, AI empowerment can significantly improve the effectiveness and transparency of internal controls through real-time data monitoring, intelligent early warnings, and process reengineering. This, in turn, enhances a company’s resource allocation efficiency and compliance levels^33^. On the other hand, as enterprises deepen their digital transformation, the internal control system’s reliance on data flow and cross-departmental collaboration continues to grow. The introduction of AI technology can facilitate a shift in internal control mechanisms from traditional passive defense to proactive prediction and dynamic optimization, effectively boosting an organization’s flexibility and resilience. Based on this, this paper uses the enterprise internal control index to measure the level of organizational control. This index is sourced from the DIBO Internal Control and Risk Management Database and is widely used in relevant domestic and international research to evaluate the soundness and effectiveness of enterprise internal control systems^43^. A higher value indicates a stronger internal control level (IC). The regression results in column (4) show a significantly positive coefficient of 0.134 at the 1% level, implying that AI enhances supply chain resilience through improvements in internal control systems. Similarly, column (5) examines the effect of internal control on resilience directly, while column (6) includes the IC index as a control variable in Model (1). In all cases, the results support the hypothesis that AI facilitates changes in internal control, which in turn strengthens supply chain resilience.

## Further analysis

### Analysis of firm heterogeneity

(1) Industry heterogeneity analysis. High-tech enterprises, thanks to their stronger innovation capabilities, higher technological intensity, and more comprehensive information infrastructure, possess a greater capacity for technology absorption and a stronger willingness for digital transformation. Aligned with Technology Acceptance Theory and Dynamic Capabilities Theory, these firms tend to adopt cutting-edge technologies like AI more quickly, integrating them into their production, supply chain management, and decision-making processes. This allows them to fully leverage AI’s benefits, such as data analysis, intelligent forecasting, and automated optimization, thereby effectively enhancing supply chain flexibility, response speed, and risk prevention and control capabilities^44^. In contrast, non-high-tech enterprises face higher barriers to new technology adoption due to limitations in capital, talent, and innovation mechanisms, which restricts the empowering effect of AI. This paper classifies sample enterprises into high-tech and non-high-tech categories based on the 2012 “Guidelines for Industry Classification of Listed Companies” revised by the Chinese government. Grouped regression results are reported in columns (1) and (2) of Table 13. The estimated coefficient for high-tech enterprises is 0.00737 and is significant at the 1% level, indicating that AI has a more pronounced effect on enhancing supply chain resilience in the high-tech manufacturing sector.

**Table 13:** Heterogeneity test regression results.

	(1)	(2)	(3)	(4)	(5)	(6)
	high-tech	Non-high-tech	downstream	Upstream	Technology-Capital	Labor
AI	0.00737^***^	−0.0145	0.0104^***^	0.00307^**^	0.00936^***^	0.000988
	(7.72)	(−1.55)	(7.00)	(2.50)	(8.49)	(0.51)
Size	0.0331	−0.257^***^	−0.0163	−0.0631	−0.0342	0.0161
	(1.10)	(−4.20)	(−0.43)	(−1.61)	(−1.15)	(0.26)
Lev	−2.456^***^	−0.888^**^	−2.263^***^	−1.852^***^	−2.427^***^	−1.052^***^
	(−13.64)	(−2.46)	(−9.61)	(−8.44)	(−13.64)	(−2.82)
Top1	−0.000716	−0.00199	−0.000879	0.000527	0.000600	−0.00476
	(−0.33)	(−0.45)	(−0.32)	(0.19)	(0.28)	(−1.07)
Board	−0.0718	0.464	0.0947	−0.150	−0.0872	0.558
	(−0.44)	(1.29)	(0.46)	(−0.70)	(−0.54)	(1.56)
FirmAge	0.0940	−0.569^**^	0.210	−0.338^**^	0.0586	−0.384
	(0.87)	(−2.45)	(1.52)	(−2.42)	(0.55)	(−1.63)
_cons	5.622^***^	11.27^***^	5.736^***^	8.643^***^	7.117^***^	4.782^***^
	(8.05)	(7.41)	(6.43)	(9.48)	(10.25)	(3.13)
Industry	Yes	Yes	Yes	Yes	Yes	Yes
Year	Yes	Yes	Yes	Yes	Yes	Yes
N	14,663	3954	10,285	8332	14,616	4001
R^2^	0.514	0.367	0.481	0.486	0.525	0.345

(2) Supply chain position analysis. Understanding the distinct positions within a supply chain is beneficial for accurately identifying weak points during risk events and implementing targeted measures. A company’s supply chain position dictates the nature of its exposure to market demand, customer relationships, and risks. Downstream enterprises, being closer to the market end, face greater pressure from demand fluctuations and changes in the external environment. According to supply chain management theory and value chain theory, downstream firms are more sensitive to environmental changes, demanding stronger capabilities for rapid response and flexible adjustment. The application of Artificial Intelligence (AI) in downstream enterprises can significantly enhance market forecasting, order management, and customer service levels, thereby strengthening their dynamic adaptability and risk resistance capabilities^15^. Following the approach of Antràs et al.^45^this study uses the 2014 China input-output table from the World Input-Output Database (WIOD) to measure the “upstreamness” of each industry. Based on this index, firms are categorized into upstream and downstream segments. Columns (3) and (4) report the regression results for downstream and upstream firms, respectively. The estimated coefficient for downstream firms is 0.00307, significant at the 1% level, while the coefficient for upstream firms is 0.0104, significant at the 5% level. These findings suggest that downstream firms benefit more substantially from AI adoption in terms of improving supply chain resilience.

(3) Factor intensity, capital and technology-intensive manufacturing enterprises typically have a stronger digital foundation and higher capacity for technology absorption. Their production processes, supply chain management, and decision-making systems rely more heavily on information technology and data-driven approaches^46^. For these firms, the implementation and integration of Artificial Intelligence (AI) are often easier, allowing AI to directly impact areas such as production automation, risk identification, and supply chain optimization. This, in turn, unlocks data value, improves response speed, and enhances resource allocation efficiency, leading to a more significant boost in supply chain resilience. In contrast, labor-intensive enterprises primarily rely on low-cost labor for their core competitiveness, and their production and management processes are relatively less sophisticated. Their ability to adopt and integrate new technologies is limited. AI empowerment in such companies often faces practical constraints like high costs, talent shortages, and organizational inertia, making it difficult to fully realize its potential in enhancing supply chain resilience. This paper, drawing on the methodology of Yin et al.^46^categorizes sample enterprises based on the median ratio of fixed assets to total assets: samples at or above the median are classified as capital-intensive enterprises. Subsequently, samples are classified based on the ratio of R&D expenditure to employee compensation: samples with a ratio greater than 1 are identified as technology-intensive enterprises. Finally, enterprises that are neither capital-intensive nor technology-intensive are classified as labor-intensive enterprises. The regression results, presented in Table 13, show that for capital and technology-intensive enterprises, the regression coefficient is 0.00936, which is significantly positive at the 1% level. This indicates that the effect of AI empowerment on enhancing supply chain resilience is more pronounced in capital and technology-intensive enterprises, while its impact is less evident in labor-intensive enterprises.

(4) Geographic Location. The level of regional economic development and the innovation environment are crucial external factors influencing an enterprise’s technology adoption and supply chain management capabilities. China’s eastern region boasts a robust economic foundation, well-developed digital infrastructure, abundant innovation resources, and strong government support, providing a superior environment for manufacturing enterprises to apply AI technology and undergo digital transformation in their supply chains^47^. Enterprises in the eastern region can more easily access high-end talent and technical services, and they can adapt more quickly to changes in the external environment, leveraging AI’s role in supply chain risk prediction and collaborative optimization to significantly enhance resilience. Conversely, manufacturing enterprises in the central and western regions face practical challenges such as weak digital infrastructure, limited flow of technology and talent, and relatively lagging policy support. The implementation of AI empowerment in these areas is more difficult and yields limited results. The regression results, shown in Table 14, indicate that the regression coefficient for the eastern region is 0.00771, which is significantly positive at the 1% level. This suggests that the enhancing effect of AI empowerment on supply chain resilience is more pronounced for enterprises in the eastern region, while it is relatively less significant in the central and western regions.

**Table 14:** Heterogeneity test regression results.

	(1)	(2)	(3)	(4)	(5)	(6)	(7)
	East	Mid-West	No Overseas Background	Overseas Background	Startup Phase	Growth Phase	Maturity Phase
AI	0.00771^***^	0.0000942	0.00735^***^	0.00133	0.00218	0.00709^***^	0.00766^***^
	(7.83)	(0.03)	(7.09)	(0.49)	(1.40)	(4.51)	(4.36)
Size	−0.0371	−0.0606	−0.0617^**^	0.0382	−0.0788	0.136^**^	−0.112^**^
	(−1.15)	(−1.16)	(−2.00)	(0.60)	(−1.53)	(2.56)	(−2.55)
Lev	−2.192^***^	−1.672^***^	−1.937^***^	−2.936^***^	−2.418^***^	−1.681^***^	−1.764^***^
	(−11.21)	(−5.54)	(−11.08)	(−6.38)	(−8.92)	(−5.65)	(−6.38)
Top1	−0.00123	0.00100	0.00184	−0.0115^**^	−0.00950^***^	−0.0104^***^	0.00857^**^
	(−0.54)	(0.25)	(0.85)	(−2.43)	(−3.33)	(−2.94)	(2.23)
Board	−0.225	0.526^*^	0.124	−0.544	−0.320	−0.0889	0.241
	(−1.29)	(1.76)	(0.74)	(−1.58)	(−1.46)	(−0.34)	(0.85)
FirmAge	0.00172	−0.0606	0.00680	−0.134	−0.349^***^	0.987^***^	0.531^*^
	(0.02)	(−0.29)	(0.06)	(−0.56)	(−2.58)	(5.10)	(1.91)
_cons	7.394^***^	6.402^***^	6.994^***^	7.750^***^	11.14^***^	−1.346	6.204^***^
	(9.88)	(5.03)	(9.72)	(5.18)	(9.49)	(−1.00)	(4.86)
Industry	Yes	Yes	Yes	Yes	Yes	Yes	Yes
Year	Yes	Yes	Yes	Yes	Yes	Yes	Yes
N	13,211	5406	15,953	2664	7015	5267	6335
R^2^	0.518	0.401	0.472	0.547	0.357	0.679	0.344

(5) Executive Overseas Background. An international background for the executive team is generally believed to enhance a company’s strategic foresight, innovation capabilities, and resource integration. However, regarding the mechanism of AI empowering supply chain resilience, domestic executive teams are often more familiar with the local market environment and policy directives. They possess a deeper understanding of the pain points and bottlenecks in local digital transformation and supply chain management, enabling them to drive the implementation of AI technology more aligned with the company’s actual needs^48^. While executives with overseas backgrounds offer an international perspective, their management experience and technology adoption logic might be better suited for developed markets. When facing AI empowerment and supply chain reforms in local manufacturing enterprises, they may experience “disconnection” or “lack of local fit,” leading to less pronounced practical effects in policy response and resource coordination compared to local executives. The regression results, as shown in Table 14, indicate that for enterprises without executives with overseas backgrounds, the regression coefficient is 0.00735, which is significantly positive at the 1% level. This suggests that the enhancement of supply chain resilience through AI empowerment is more significant in enterprises led by executives without overseas backgrounds, while it is not significant in enterprises with executives possessing overseas backgrounds.

(6) Enterprise Life Cycle. The life cycle stage an enterprise is in determines its resource base, management capabilities, and technology absorption capacity^49^. Start-up enterprises, being small with limited resources and incomplete management systems, lack the ability to absorb and integrate emerging technologies like AI, often failing to achieve the expected effects of AI empowerment on supply chain resilience. In contrast, growth-stage and mature enterprises, having undergone scale expansion and capability accumulation, possess more complete information systems and management mechanisms. They are better positioned to leverage AI for production optimization, risk control, and supply chain collaboration, thereby significantly enhancing supply chain resilience. The regression results, as shown in Table 14, indicate that the regression coefficients for both growth-stage and mature enterprises are significantly positive at the 1% level. In summary, the enhancement of supply chain resilience through AI empowerment is primarily evident in growth-stage and mature enterprises, with the effect being insignificant in start-up enterprises.

## Retesting based on AI decomposition

In this study’s variable construction, Artificial Intelligence (AI) is divided into three levels: AI Terminology, AI Technology, and AI Application. First, the AI Terminology indicator primarily measures the frequency and intensity of AI-related expressions in corporate annual reports and other textual documents. This is used to reflect the enterprise’s strategic-level attention to AI and its emphasis at the “expressive level,” helping to identify whether the enterprise incorporates AI into its development strategy or as a key component of brand promotion. Second, the AI Technology indicator focuses on the enterprise’s actual R&D investment and technological reserves in core AI algorithms, data modeling, and technology platforms. This reflects the depth of the enterprise’s capabilities in AI technology development and innovation. Finally, the AI Application indicator concentrates on the practical implementation of AI technology in various enterprise scenarios, such as actual business operations, supply chain management, production optimization, and risk control. This demonstrates the degree of deep integration between the AI strategy and enterprise operations.

Each of these three AI variable types was incorporated into the regression model to systematically examine enterprises’ actual performance across different AI empowerment paths and their impact on supply chain resilience. The regression results, presented in Table 15, show that the regression coefficients for AI Terminology, AI Technology, and AI Application are 0.00743, 1.048, and 0.0113, respectively, and all are significantly positive at the 1% level. This result indicates that manufacturing enterprises not only highly value AI in their strategic discourse but also that their efforts in technology R&D and practical application have effectively promoted the enhancement of supply chain resilience. Simultaneously, it also reflects that China’s manufacturing enterprises still have room for further improvement in deep AI technology development and practical application.

**Table 15:** Further analysis of regression results.

	(1)	(2)	(3)
	SCR	SCR	SCR
AI	0.00743^***^	1.048	0.0113^**^
	(7.00)	(1.18)	(2.44)
Size	−0.0369	−0.0389	−0.0356
	(−1.35)	(−1.42)	(−1.30)
Lev	−2.068^***^	−2.030^***^	−2.027^***^
	(−12.69)	(−12.45)	(−12.44)
Top1	−0.000152	−0.000131	−0.000293
	(−0.08)	(−0.07)	(−0.15)
Board	0.0223	0.0194	0.0157
	(0.15)	(0.13)	(0.10)
FirmAge	−0.0397	−0.0583	−0.0427
	(−0.40)	(−0.59)	(−0.43)
_cons	6.978^***^	7.124^***^	6.980^***^
	(10.87)	(11.09)	(10.82)
Industry	Yes	Yes	Yes
Year	Yes	Yes	Yes
N	18,617	18,617	18,617
R^2^	0.479	0.478	0.478

## Conclusion and insights

Artificial Intelligence (AI), as an emerging technology at the forefront of the new wave of technological revolution and industrial transformation, holds significant potential to drive organizational change and foster supply chain resilience. However, due to methodological limitations and data availability constraints, there remains a lack of consensus on how AI contributes to enhancing supply chain resilience at the micro (firm) level, particularly regarding the role and mechanism of organizational change in this process. Drawing on panel data from A-share listed manufacturing firms in China over the period 2013–2022, this study employs text analysis to construct firm-level AI indicators and applies factor analysis—guided by the theory of core competence—to develop a multidimensional measure of supply chain resilience across five dimensions. Based on both theoretical reasoning and empirical evidence, the study arrives at the following conclusions:

(1) The higher the degree of AI empowerment of manufacturing enterprises, the more obvious the supply chain toughness improvement.

(2) The mechanism test shows that AI can achieve the improvement of manufacturing supply chain toughness by strengthening organizational structure change and internal control change.

(3) Heterogeneity analysis reveals that the enhancing effect of Artificial Intelligence on supply chain resilience varies significantly across different supply chain positions, factor intensities, regional locations, industry characteristics, executive overseas backgrounds, and enterprise life cycles.

(4) Further analysis shows that the effective combination of AI terminology and application level in manufacturing enterprises significantly improves supply chain resilience.

Based on the above research findings, this paper proposes the following policy recommendations:

(1) In the face of the growing complexity and uncertainty within the global manufacturing supply chain, businesses must firmly advance strategic transformation empowered by artificial intelligence. Countries should consider implementing tailored incentive measures based on their unique supply chain characteristics. These could include increased financial subsidies, specific tax reductions, and optimized talent acquisition policies, all aimed at accelerating corporate investment and innovation in the field of AI. Additionally, efforts should be made to attract and cultivate AI R&D talent—particularly in technological domains where development bottlenecks in China remain significant. The government is encouraged to establish dedicated funding programs to support foundational technological breakthroughs. Such initiatives would help mitigate the risk of “chokepoints” (i.e., technology dependency risks) in the integration of AI within manufacturing, thereby reducing potential vulnerabilities in the supply chain.

(2) Encourage supply chain enterprises to proactively adapt and optimize their organizational structures in response to technological advancements. Companies worldwide should actively adapt to the trends of digitalization and intelligent transformation, exploring organizational adjustment paths that suit their unique needs. At the policy level, governments can support this transformation by offering targeted subsidies and strategic guidance to promote flatter organizational hierarchies and facilitate interdepartmental collaboration and information flow. Leveraging AI’s capabilities in data analysis and demand forecasting can help improve enterprise-level production and personnel management, dismantle traditional organizational silos, and reduce excessive hierarchical layers—thus enhancing overall decision-making efficiency. Furthermore, by applying advanced algorithms and predictive models, enterprises can more effectively identify and respond to potential risks, promote employee participation in decision-making, and strengthen organizational flexibility and responsiveness in a dynamic environment.

(3) Guiding Intelligent Supply Chain Development through Enterprise Collaboration and Regional Synergy. The enhancement of supply chain resilience relies not only on technology itself but also on differentiated collaborative development among various types of enterprises within the supply chain. Targeted coordination mechanisms should be established for different segments of the supply chain—for instance, downstream enterprises can focus on market responsiveness and risk monitoring, while upstream enterprises should emphasize resource integration and supply assurance to improve the overall adaptability of the network. At the same time, the distinct characteristics of enterprises—such as factor intensity and life cycle stage—must be fully considered. Capital- and technology-intensive enterprises, along with those in the growth or maturity stages, typically possess stronger capacities for technology adoption and collaboration, and can take the lead in promoting AI-driven deep data sharing and intelligent early warning platforms. In contrast, labor-intensive and start-up enterprises should prioritize the enhancement of their basic digital capabilities and the cultivation of collaborative mechanisms. Policy frameworks should encourage diverse types of enterprises to jointly improve resilience through the flow of data elements, knowledge transfer, and platform-based cooperation. Regional heterogeneity must also be acknowledged: eastern regions may take the lead in building intelligent supply chain collaboration networks, while central and western regions should focus on improving infrastructure and cross-regional coordination mechanisms to achieve national and even global supply chain connectivity. The international background of executive teams also influences collaboration efficiency and innovation paths. Therefore, fostering communication among management teams with both local and global perspectives is essential for promoting diverse collaboration and the sharing of best practices. By designing layered and categorized coordination mechanisms, supply chain integration across production, supply, and sales can be effectively guided, thereby enhancing the overall resilience and risk resistance of the system.

(4) Promoting Inclusive AI Transformation Across Diverse Types of Enterprises. Enterprises face varying challenges in leveraging AI to enhance supply chain resilience—particularly non-high-tech firms, labor-intensive businesses, enterprises located in central and western regions, startups in their early stages, and those with less internationally experienced executive teams. These entities often encounter constraints in funding, talent, and technological foundations. It is therefore recommended that policymakers and industry associations collaborate with universities and research institutions to establish multi-tiered platforms for technical consulting and innovation resource sharing, thereby advancing the widespread adoption of AI and promoting deeper integration of industry, academia, and research. Given the heterogeneity in factor intensity and enterprise life cycle stages, capital- and technology-intensive enterprises, as well as those in the growth or maturity phases, should accelerate the integration of core AI technologies and foster application-driven innovation. Meanwhile, labor-intensive and start-up enterprises need to focus on skills training and reducing technological barriers to entry. In terms of regional disparities, the eastern region should continue to lead AI transformation efforts, while central and western regions should prioritize the development of digital infrastructure and the attraction of skilled talent. Differences in executive backgrounds should also be addressed by encouraging the introduction of diverse management teams and enhancing digital awareness at the leadership level. Overall, differentiated policy instruments should be formulated to support an inclusive and sustainable AI transformation across industries, regions, supply chain segments, and enterprise types—ensuring that the benefits of AI-driven supply chain resilience are equitably shared throughout society.

### Limitations and future research directions

Despite the contributions of this study in constructing a mechanism model of how artificial intelligence enhances supply chain resilience in manufacturing firms, several limitations remain. First, the sample is based on Chinese listed manufacturing firms, which may limit the generalizability of the findings to small- and medium-sized enterprises or less technology-intensive firms. Future research could expand the sample scope to conduct cross-industry or cross-firm-type comparative studies. Second, some mediating variables are measured using proxy indicators. For instance, the proportion of executives is used to represent organizational structural transformation. While theoretically acceptable, this proxy has limited explanatory power in capturing complex changes in decision-making and coordination mechanisms. Future studies may incorporate more granular executive characteristics or internal governance data to improve measurement accuracy. Lastly, this research focuses on firm-level data, which restricts insights into the micro-level decision-making processes or multi-level interactions. Further studies could adopt multi-level modeling, case studies, or behavioral experiments to explore the nuanced pathways through which AI enables supply chain resilience.

## Data Availability

Some or all data, models, or codes generated or used during the study are available from the corresponding author by request.

## References

[1] Massari, G. F., Nacchiero, R. & Giannoccaro, I. Transformative supply chains: the enabling role of digital technologies. *Int. J. Prod. Econ.* **283**, 109562 (2025).

[2] Dey, P. K. et al. Artificial Intelligence-driven supply chain resilience in Vietnamese manufacturing Small-and Medium-sized enterprises. *Int. J. Prod. Res.* **62** (15), 5417–5456 (2024).

[3] Davenport, T. H. & Ronanki, R. Artificial intelligence for the real world. *Harvard Business Rev.* **96** (1), 108–116 (2018).

[4] Weber, M., Engert, M., Schaffer, N., Weking, J. & Krcmar, H. Organizational capabilities for Ai implementation—co** with inscrutability and data dependency in Ai. *Inform. Syst. Front.* **25** (4), 1549–1569 (2023).

[5] Polas, M. R. H. et al. A journey from traditional supply chain processes to Sustainability‐Oriented blockchain supply chain: the critical role of organizational capabilities. *Bus. Strategy Environ.* **34** (3), 3522–3543 (2025).

[6] Belhadi, A. et al. Building Supply-chain resilience: an artificial Intelligence-Based technique and Decision-making framework. *Int. J. Prod. Res.* **60** (14), 4487–4507 (2022).

[7] Ralston, P. & Blackhurst, J. Industry 4.0 and resilience in the supply chain: A driver of capability enhancement or capability loss?? *Int. J. Prod. Res.* **58** (16), 5006–5019 (2020).

[8] Zhang, S. & Gu, C. Supply chain digitalization and supply chain resilience. *Finance Econ. Res.* **50** (07), 21–34 (2024).

[9] Ponomarov, S. Y. & Holcomb, M. C. Understanding the concept of supply chain resilience. *Int. J. Logistics Manage.* **20** (1), 124–143 (2009).

[10] Agyabeng-Mensah, Y., Oloruntoba, R., Earnest, J. & Mohammadi, H. *Sustainable Supply Chain Management and Performance Outcomes: Supply Chain Practice View and Mediated Moderation Perspectives* (Business Strategy and the Environment, 2025).

[11] Shi, X., Prajogo, D., Fan, D. & Oke, A. Is operational flexibility a viable strategy during major supply chain disruptions? Evidence from the COVID-19 pandemic. *Transp. Res. E*. **195**, 103952 (2025).

[12] Dutta, P. et al. Blockchain technology in supply chain operations: applications, challenges and research opportunities. *Transp. Res. E*. **142**, 102067 (2020). 10.1016/j.tre.2020.102067 PMC7522652 33013183

[13] Bassiouni, M. M. et al. Deep learning approaches to identify order status in A complex supply chain. *Expert Syst. Appl.* **250**, 123947 (2024).

[14] Brusset, X. & Teller, C. Supply chain capabilities, risks, and resilience. *Int. J. Prod. Econ.* **184**, 59–68 (2017).

[15] Christopher, M. & Peck, H. Building the resilient supply chain. *Int. J. Logistics Manage.* **15** (2), 1–13 (2004).

[16] Kumar, G., Subramanian, N. & Arputham, R. M. Missing link between sustainability collaborative strategy and supply chain performance: role of dynamic capability. *Int. J. Prod. Econ.* **203**, 96–109 (2018).

[17] Niu, B., Lai, C., Zheng, Z., Zeng, F. & Dai, Z. Will supplier’s quality improvement discourage competing buyers’ joint procurement? Impact of product differentiation and manufacturing Cooperation. *Int. J. Prod. Econ.* **286**, 109666 (2025).

[18] Ding, L. et al. Exploring the application pattern of generative intelligence in new power systems based on large Language models. *Autom. Electr. Power Syst.* **48** (19), 1–13 (2024).

[19] Nussibaliyeva, A. et al. Development of an artificial vision for A parallel manipulator using Machine-to-Machine technologies. *Sensors* **24** (12), 3792 (2024). 38931576 10.3390/s24123792 PMC11207595

[20] Lerch, C. M. et al. AI-readiness and production resilience: empirical evidence from German manufacturing in times of the Covid-19 pandemic. *Int. J. Prod. Res.* **62** (15), 5378–5399 (2024).

[21] Tang, H., Fang, S. & Jiang, D. Market performance of digital transformation: can digital M&A enhance market power of manufacturing firms?? *J. Quant. Tech. Econ.* **39** (12), 90–110 (2022).

[22] Melville, N., Kraemer, K. & Gurbaxani, V. Information technology and organizational performance: an integrative model of IT business value. *MIS Q.* **28** (2), 283–322 (2004).

[23] Cannavacciuolo, L., Ferraro, G., Ponsiglione, C., Primario, S. & Quinto, I. Technological innovation-enabling industry 4.0 paradigm: A systematic literature review. *Technovation* **124**, 102733 (2023).

[24] Ivanov, D. & Dolgui, A. Viability of intertwined supply networks: extending the supply chain resilience angles towards survivability. A position paper motivated by COVID-19 outbreak. *Int. J. Prod. Res.* **58** (10), 2904–2915 (2020).

[25] Peterson, N. A., Lowe, J. B. & Aquilino, M. L. Linking social cohesion and gender to intrapersonal and interactional empowerment: support and new implications for theory. *J. Community Psychol.* **33** (2), 233–244 (2005).

[26] Chen, Y., Yang, S., Zhang, Z. & Shen, H. How does digital enablement affect product customization? The roles of innovation capability and network embeddedness. *Technol. Forecast. Soc. Chang.* **201**, 123272 (2024).

[27] Qi, Y. D. & Xiao, X. Enterprise management transformation in the digital economy era. *Manage. World*. **36** (06), 135–152 (2020).

[28] Li, C. et al. Artificial intelligence, resource reallocation, and corporate innovation efficiency: evidence from china’s listed companies. *Resour. Policy*. **81**, 103324 (2023).

[29] Zhao, Y. *Job Relatedness, Friend Network Scope, Network Status and Job Meaning: A Moderated Mediation Model*37111–124 (Science of Science and Management of S&T, 2016). 12.

[30] Sun, S. Y. & Wang, Y. L. *The Diminishing Effect of Perceived Self-serving Leadership Empowerment on Upward Support: Based on Self-enhancement Theory*1–21 (Foreign Economics & Management, 2024).

[31] Jöhnk, J., Weißert, M. & Wyrtki, K. Ready or not, AI comes—an interview study of organizational AI readiness factors. *Bus. Inform. Syst. Eng.* **63** (1), 5–20 (2021).

[32] Huo, L. & Zhang, L. Y. AI-driven modernization of china’s industrial chains. *J. Northwest. Univ. (Philosophy Social Sci. Edition)*. **54** (04), 86–102 (2024).

[33] Zhang, X. et al. Digital manufacturing collaboration in High-end equipment under new generation information technology. *Manage. World*. **39** (01), 190–204 (2023).

[34] Ashraf, M. The role of peer events in corporate governance: evidence from data breaches. *Acc. Rev.* **97** (2), 1–24 (2022).

[35] Acemoglu, D. & Restrepo, P. The race between man and machine: implications of technology for growth, factor shares, and employment. *Am. Econ. Rev.* **108** (6), 1488–1542 (2018).

[36] Lu, S. Supply chain coordination: an analysis based on core competency theory. *Enterp. Econ.* **11**, 56–58 (2008).

[37] Han, Z. & Gao, X. Mixed-ownership reform of state-owned enterprises, industrial linkage, and climbing the global value chain: A mechanism analysis based on the TOE framework. *Finance Econ. Res.* **9**, 56–71 (2024).

[38] Chen, J., Guo, Z. & Lei, Z. Research on the mechanisms of the digital transformation of manufacturing enterprises for carbon emissions reduction. *J. Clean. Prod.* **449**, 141817 (2024).

[39] Xiao, L., Xu, X., Xue, W. & Lei, T. A study on the antecedents of enterprise inventory agility under the COVID-19 pandemic: an exploration based on the AMC framework. *J. Industrial Eng. Eng. Manage.* 1–17. 10.13587/j.cnki.jieem.2025.05.018 (2025).

[40] Bárcena-Ruiz, J. C., Garzón, M. B. & Sagasta, A. Environmental corporate social responsibility, R&D and disclosure of green innovation knowledge. *Energy Econ.* **120**, 106628 (2023).

[41] Yao, J. Q. et al. How can AI improve enterprise productivity?? A perspective based on labor skill structure adjustment. *Manage. World*. **40** (02), 101–116 (2024).

[42] Li, Y., Bai, T., Sha, Y. & Xu, Z. Concentration of supply chain, internal control, and corporate risk-taking. *Int. Rev. Financial Anal.* **103**, 104261 (2025).

[43] Choi, A., Lee, W. J., Lee, Y. G. & Zhou, G. Internal information quality and corporate employment decisions. *Australian Acc. Rev.* **33** (3), 262–283 (2023).

[44] Babina, T., Fedyk, A., He, A. & Hodson, J. Artificial intelligence, firm growth, and product innovation. *J. Financ. Econ.* **151**, 103745 (2024).

[45] Antràs, P. et al. Measuring the upstreamness of production and trade flows. *Am. Econ. Rev.* **102** (3), 412–416 (2012).

[46] Yin, M., Sheng, L. & Li, W. Executive incentives, innovation input, and firm performance: an industry-based empirical study from the perspective of endogeneity. *Nankai Bus. Rev.* **21** (1), 109–117 (2018).

[47] Wang, Q., Zhang, F. & Li, R. Artificial intelligence and sustainable development during urbanization: perspectives on AI R&D innovation, AI infrastructure, and AI market advantage. *Sustain. Dev.* **33** (1), 1136–1156 (2025).

[48] Han, W., Zhu, W., Song, Z. & Lu, R. Innovative resources driven artificial intelligence orientation: the moderating role of environmental and executives’ characteristics. *Technol. Soc.* **81**, 102837 (2025).

[49] Lee, C. C., Zou, J. & Chen, P. F. The impact of artificial intelligence on the energy consumption of corporations: the role of human capital. *Energy Econ.* **143**, 108231 (2025).

